# Ca_1__−*x*_Li*_x_*Al_1__−_*_x_*Si_1+*x*_N_3_:Eu^2+^ solid solutions as broadband, color-tunable and thermally robust red phosphors for superior color rendition white light-emitting diodes

**DOI:** 10.1038/lsa.2016.155

**Published:** 2016-10-21

**Authors:** Le Wang, Rong-Jun Xie, Yuanqiang Li, Xiaojun Wang, Chong-Geng Ma, Dong Luo, Takashi Takeda, Yi-Ting Tsai, Ru-Shi Liu, Naoto Hirosaki

**Affiliations:** 1 College of Optics and Electronic Science and Technology, China Jiliang University, Hangzhou, Zhejiang 310018, China; 2 College of Materials, Xiamen University, Xiamen, Fujian 361005, China; 3 Sialon Group, National Institute for Materials Science (NIMS), Tsukuba, Ibaraki 305-0044, Japan; 4 Intematix Corporation, Fremont, CA 94538, USA; 5 College of Sciences, Chongqing University of Posts and Telecommunications, Chongqing 400065, China; 6 Department of Chemistry, Taiwan University; 7 Department of Mechanical Engineering and Graduate Institute of Manufacturing Technology, Taipei University of Technology

**Keywords:** color rendering, LEDs, nitride, phosphor, structure disorder

## Abstract

Color rendition, luminous efficacy and reliability are three key technical parameters for white light-emitting diodes (wLEDs) that are dominantly determined by down-conversion phosphors. However, there is usually an inevitable trade-off between color rendition and luminescence efficacy because the spectrum of red phosphor (that is, spectral broadness and position) cannot satisfy them simultaneously. In this work, we report a very promising red phosphor that can minimize the aforementioned trade-off via structure and band-gap engineering, achieved by introducing isostructural LiSi_2_N_3_ into CaAlSiN_3_:Eu^2+^. The solid solution phosphors show both substantial spectra broadening (88→117 nm) and blueshift (652→642 nm), along with a significant improvement in thermal quenching (only a 6% reduction at 150 °C), which are strongly associated with electronic and crystal structure evolutions. The broadband and robust red phosphor thus enables fabrication of super-high color rendering wLEDs (Ra=95 and R9=96) concurrently with the maintenance of a high-luminous efficacy (101 lm W^−1^), validating its superiority in high-performance solid state lightings over currently used red phosphors.

## Introduction

White light-emitting diodes (wLEDs) are broadly known as one of most efficient and environmental friendly lighting technology, greatly contributing to energy saving and greenhouse gas reduction^1, 2^. They are now gradually replacing traditional incandescent bulbs and fluorescence tubes for general lighting, and their luminous efficacy, color rendition and reliability are key factors determining these replacements. For those wLEDs using a single garnet yellow phosphor (Y_3_Al_5_O_12_:Ce^3+^), the insufficient red component in the spectra leads to a small color rendering index (Ra<80), making them unsuitable for high-quality general lighting^3^. A single Eu^2+^-doped oxychloride white-light phosphor was reported to produce high color rendering wLEDs (Ra=91, R9=90.2), but unfortunately, it cannot be excited by blue LEDs^4^. Recently, Pan discovered a broadband yellow phosphor (Ba_0.93_Eu_0.07_Al_2_O_4_) with an enhanced red spectral component, which yields a medium color rendition (Ra~85)^5^. However, to achieve much higher color rendering indices, a red phosphor is thus essentially required to enhance the red spectral part^6, 7, 8^. Currently, several promising red-emitting phosphors have been proven to effectively enhance the color rendition, including (Ca, Sr, Ba)_2_Si_5_N_8_:Eu^2+^ (ref. 9), (Sr, Ca)AlSiN_3_:Eu^2+^ (refs 10, 11), SrLiAl_3_N_4_:Eu^2+^ (ref. 12) and K_2_(Si, Ti)F_6_:Mn^4+^ (refs 8, 13).

In general, multi-phosphor-converted wLEDs have higher color rendition (Ra>80) but lower luminous efficacy (~70%) than one-phosphor-converted wLEDs^14^. This indicates that there exists a fundamental trade-off relation between color rendering index and luminous efficacy, which means that improvements in one generally coincide with diminishment of the other. Moreover, even for the color rendering index, except for the Ra value (the average of the first eight color rendering indices), the ninth color rendering index (R9, the red content) has received much attention because reds are everywhere: there is much red in the color of human skin and of meat, fruits and vegetables, clothes and so on. The R9 value is always negative for one-phosphor-converted wLEDs, and increases largely by complementing a red phosphor^3, 7, 12^. Kimura^7^ combined a phosphor blend of BaSi_2_O_2_N_2_:Eu^2+^, β-sialon:Eu^2+^, Ca-α-sialon:Eu^2+^ and CaAlSiN_3_:Eu^2+^ with a blue LED and fabricated high-rendition wLEDs with Ra=95–98, R9=89 and a luminous efficacy of 28–35 lm W^−1^. Pust^12^ used a narrow-band red phosphor (SrLiAl_3_N_4_:Eu^2+^) instead of the broadband CaAlSiN_3_:Eu^2+^ to enhance the luminous efficacy of wLEDs (14% increase), but sacrificed both Ra (=81) and R9 (=57) values. Brinkley^15^ reported a three-band wLED using the combination of YAG:Ce^3+^ and a short-wavelength red Sr_2_Si_5_N_8_:Eu^2+^ phosphor. The luminous efficacy was sufficiently high (94 lm W^−1^), but Ra (~72) still needs improvement for general lighting.

As mentioned above, the narrow-band or short-wavelength (blueshifted) red phosphors are able to attain high-luminous efficacy but also decrease color rendering indices (typically R9). To eliminate or minimize this trade-off, there is a need for the red phosphor to have both a broadband and blueshifted emission simultaneously. CaAlSiN_3_:Eu^2+^ is a deep-red phosphor and superior to its counterparts, such as Sr_2_Si_5_N_8_:Eu^2+^ and K_2_SiF_6_:Mn^4+^, in thermal stability, reliability and quantum efficiency^16, 17^. The color tuning of CaAlSiN_3_:Eu^2+^ can be achieved by Sr→Ca substitution. This leads to a substantial blueshift in emission (650→610 nm), but concurrently, an unfortunate dramatic narrowing in the full width at half maximum (FWHM) of the emission spectrum (94→75 nm)^11, 18^. Recently, Huang and colleagues^19^ observed emission spectral broadening in CaAlSiN_3_:Eu^2+^ by co-doping with C and Al to form CaAl_1__−__4*δ*/3__−_*_x_*Si_1+*δ*+*x*_N_3__−*x*_C*_x_*. However, the mechanism of the spectral broadening remains elusive.

Structural disorder is often recognized as an origin of the spectral broadening in luminescent materials and can be created by introducing impurities in the crystal lattice^20, 21^. CaAlSiN_3_ is isostructural with LiSi_2_N_3_, both crystallizing in an orthorhombic Cmc2_1_ structure, which makes it possible to introduce LiSi_2_N_3_ into CaAlSiN_3_ as an ‘impurity’^22^. The introduction can also be considered as the double-substitution in CaAlSiN_3_, that is, [Li,Si]^5+^→[Ca,Al]^5+^. Silicon and aluminum randomly and equally reside at the same crystallographic site in the CaAlSiN_3_ lattice; thus, the double-substitution will increase the Si/Al ratio and in turn result in an increase in the degree of structural disorder. The broadening of the emission spectrum is therefore anticipated. Moreover, the solid-solution formation between CaAlSiN_3_ and LiSi_2_N_3_ (Ca_1__−_*_x_*Li*_x_*Al_1__−_*_x_*Si_1+*x*_N_3_) will also definitely change the electronic and crystal structure of the host crystals, thus having a great influence on photoluminescence properties, such as the spectral tuning, luminescence efficiency and thermal quenching.

In this work, we report, for the first time, the realization of simultaneous spectral broadening and blueshift in CaAlSiN_3_:Eu^2+^ by forming solid solutions via double cationic substitutions. The newly developed broadband red phosphors exhibit a high external quantum efficiency (70–78%) and enhanced thermal stability, enabling them to be superior to the commonly used Ca_1__−__*x*_Sr*_x_*AlSiN_3_:Eu^2+^ in reliability and color rendition. We demonstrate that by using the Ca_1__−*x*_Li*_x_*Al_1__−*x*_Si_1+*x*_N_3_:Eu^2+^ (*x*=0.20) red phosphor, a super-high color rendering index (Ra=95 and R9=96) can be achieved without compromising the luminous efficacy (~101 lm W^−1^) of wLEDs.

## Materials and methods

The phosphor powders of Ca_1__−*x*_Li*_x_*Al_1__−*x*_Si_1+*x*_N_3_:Eu^2+^ were prepared by using a gas pressure sintering furnace. The *x* value was varied in the range of 0–0.22. Appropriate amounts of high purity Ca_3_N_2_, Si_3_N_4_, AlN, Li_3_N and EuN powders were mixed in a nitrogen-filled glove box (H_2_O<1 ppm, O_2_<1 ppm). The powders were put into BN crucibles and fired at 1800 °C for 2 h under 1.0 MPa nitrogen gas. The weak reducing atmosphere in the furnace, using graphite heating units, enabled the reduction of Eu^3+^ (EuN) into Eu^2+^ in the phosphor. After firing, the phosphor powders were pulverized by hand using a silicon nitride mortar and pestle, and further washed in deionized water at 60 °C.

The chemical composition was measured by using an inductively coupled plasma-mass spectrometer (ICP-MS, Thermo Fisher Scientific K.K., Yokohama, Japan). The nitrogen and oxygen content was measured via the selective hot-gas extraction method (TC-436, CS-444LS, LECO CO., Tokyo, Japan). The microstructure of powders was imaged using a scanning electron microscope (S-5000; Hitachi Ltd., Tokyo, Japan).

The crystal structure was determined via X-ray powder diffraction (XRD; RINT Ultima-III, Rigaku Co., Tokyo, Japan) with Cu Kα radiation (*λ*=1.54056 Å). The current and cathode voltage were 40 mA and 40 kV, respectively. The data of CaAlSiN_3_ single crystals were utilized as an initial mode for the Rietveld refinement using the GSAS package.

The valence state of Eu ions in phosphors was measured by using an X-ray absorption fine structure, and was recorded at the BL37XU beamline of the SPring-8 synchrotron radiation facility (Hyogo, Japan).

The ^29^Si- and ^7^Li- MAS nuclear magnetic resonance (NMR) spectra were collected using a 14.1-T wide-bore Bruker Advance III spectrometer (Karlsruhe, Germany). A 4-mm MAS probe was used for ^29^Si, with the sample spinning at 13.5 kHz, and a 3.2-mm probe was used for ^7^Li, with the sample spinning at 10 kHz. The Larmor frequencies for ^7^Li and ^29^Si were 233.3 and 119.2 MHz, respectively. The excitation pulse was set as 1.7 μs (the π/6-pulse) for ^7^Li, and 2.5 μs (the π/4-pulse) for ^29^Si. The recycle delay was 2 and 60 s for ^7^Li and ^29^Si, respectively.

The diffusive reflection spectrum was obtained by using a UV-Vis spectrophotometer with an integrating sphere (JASCO, Ubest V-560, Tokyo, Japan). The Spectralon diffusive white standard was used for calibration. The luminescence spectra were recorded by using a fluorescent spectrophotometer (F-4500, Hitachi Ltd., Tokyo, Japan). A 200 W Xe lamp was used as an excitation source. Quantum efficiencies were measured by using an intensified multichannel spectrometer (MCPD-7000, Otsuka Electronics, Tokyo, Japan) and computed by using the equations proposed in the literature^23^. An ultrahigh vacuum SEM with a Gemini electron gun (Omicron, Bavaria, Germany) equipped with a cathodoluminescence (CL) system was used to measure CL spectra at room temperature^24^. The diameter of the electron beam was in the order of 10 nm. The specimen was irradiated for 1 h under electron beams of 5 kV and 1000 pA before measurements.

Thermal quenching was evaluated using the MCPD-7000 by heating the phosphor up to 250 °C at a heating rate of 100 °C min^−1^ and holding at each temperature for 5 min. The high-temperature quantum efficiency was measured using a quantum yield spectrophotometer (QE-1100, Otsuka Electronics).

The prototype warm wLEDs were fabricated using a Chip-on-Board packaging solution by pumping the red Ca_1__−_*_x_*Li*_x_*Al_1__−_*_x_*Si_1+*x*_N_3_:Eu^2+^ (*x*=0 and 0.20) and commercial garnet green (LuAG or GYAG) phosphors using a blue InGaN LED chip (450 nm). Dow Corning@ OE2140 (Tokyo, Japan) was used as the epoxy resin for binding phosphors. The optical properties of these wLEDs were recorded using a spectroradiometer (LHS-1000, Everfine Co., Hangzhou, China). The spatial radiation spectrum was obtained by using a goniophotometer (LED626, Everfine Co., Hangzhou, China). wLEDs were driven at 60 mA and 2.925 V.

## Results and discussion

### Structural evolutions

Measured using inductively coupled-plasma and an oxygen/nitrogen analyzer, the synthesized samples have actual chemical compositions very similar to the nominal ones, except that half of the Li content was evaporated at high temperatures (Supplementary Table S1). Moreover, a small amount of oxygen (equal for all samples) was detected due to the contamination of the raw nitride materials.

**Figure 1:**
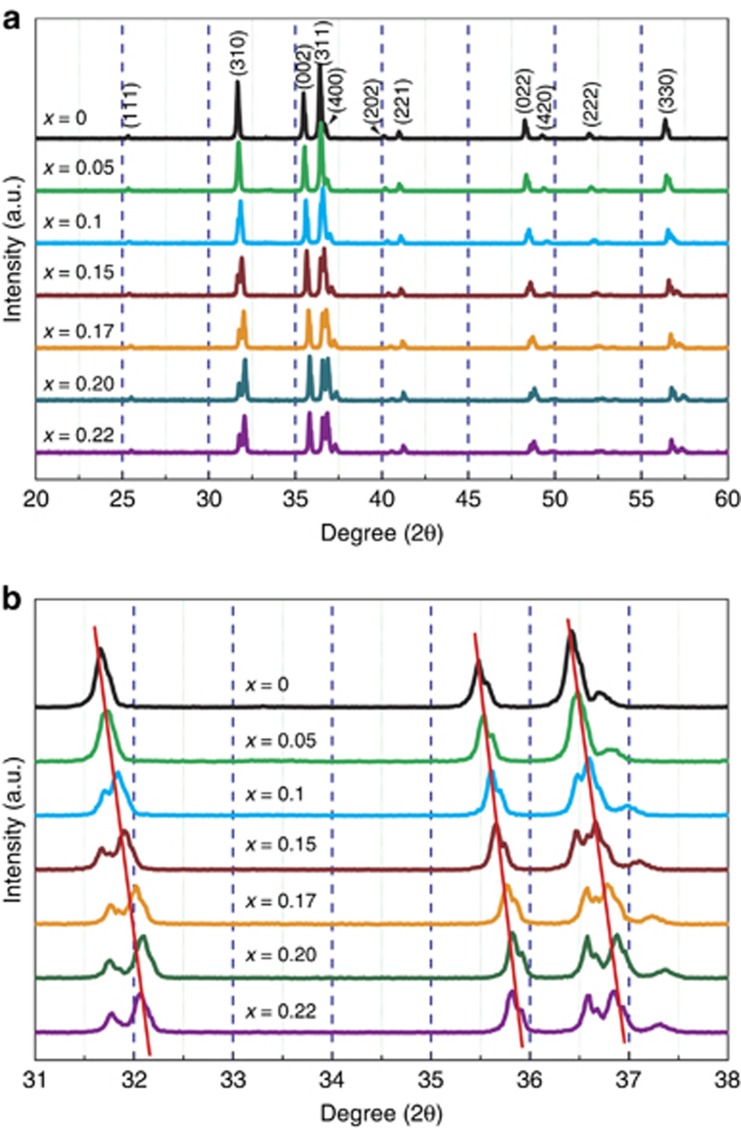
(**a**) XRD patterns of samples of Ca_1__−_*_x_*Li*_x_*Al_1__−*x*_Si_1+*x*_N_3_ with varying *x* and (**b**) enlarged XRD patterns of the portion marked in **a**, showing the diffraction peak splitting with increasing *x*.

The XRD spectra of samples are illustrated in Figure 1. All of the diffraction peaks are identified as the CaAlSiN_3_ phase, demonstrating the production of a solid solution between CaAlSiN_3_ and LiSi_2_N_3_ in all compositions (*x*=0–0.22). The XRD peaks shift toward higher angles with increasing *x*, implying lattice shrinkage owing to the smaller Li^+^ (0.76 Å, CN=6) and Si^4+^ (0.26 Å, CN=4) ions compared with Ca^2+^ (1.00 Å, CN=6) and Al^3+^ (0.39 Å, CN=4)^25^. Moreover, as seen in Figure 1b, the XRD peaks are significantly split as the LiSi_2_N_3_ content increases, indicative of the enhanced degree of structural disorder and lowered symmetry in the solid-solution phase. As we know, the structure of CaAlSiN_3_ can be considered a distorted AlN-like wurtzite superstructure, in which Al and Si are randomly distributed and disordered on the 8b site^10, 22^. The Si/Al ratio in the lattice becomes larger with the introduction of LiSi_2_N_3_ and hence further increases the structural disorder. The decrease in structural symmetry is supported by the lattice energy calculations by Vienna Ab initio simulation package (VASP)^26, 27^, which show that the monoclinic Cc structure may be more energetically stable than the orthorhombic structure for *x*=0.2 (Supplementary Fig. S1). In this work, we observed that the structure transition occurs at *x*≥0.15.

**Figure 2:**
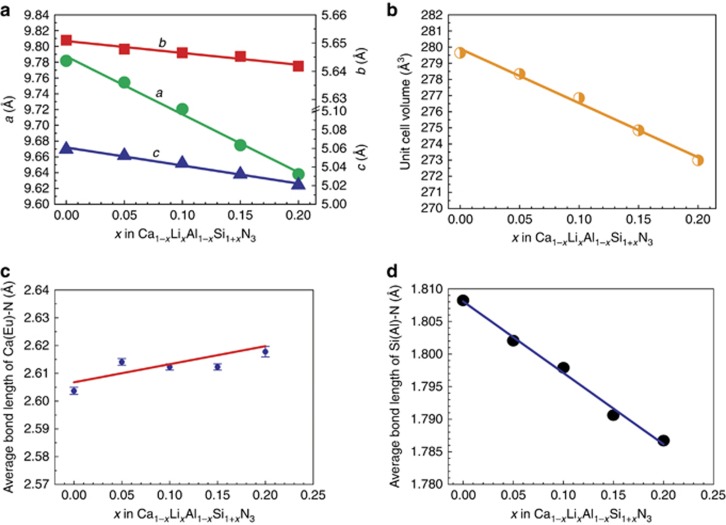
Effect of solubility of LiSi_2_N_3_ (*x* value) on (**a**) lattice constants, (**b**) cell volume, (**c**) the Ca(Eu)–N distance and (**d**) the (Si,Al)–N distance of CaAlSiN_3_:Eu^2+^.

**Figure 3:**
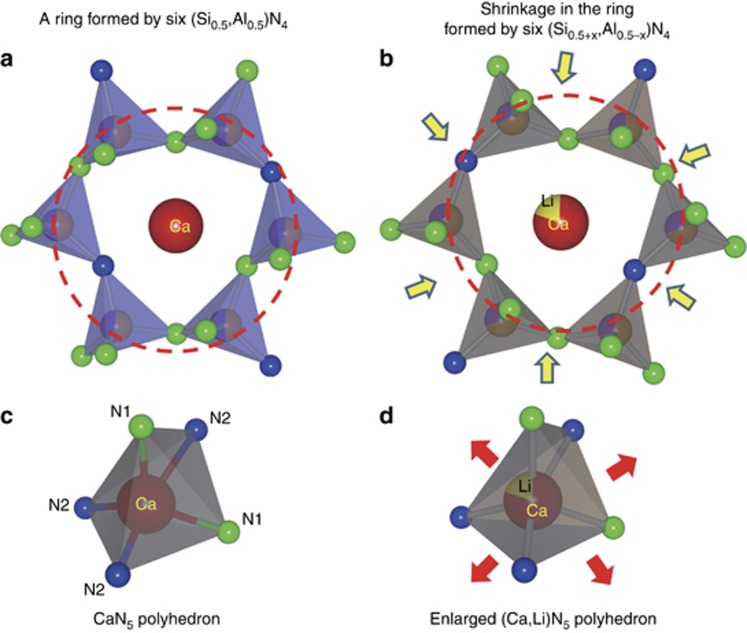
Schematics of the second coordination spheres of (**a**) CaAlSiN_3_ and (**b**) CaAlSiN_3_–LiSi_2_N_3_; the polyhedron of (**c**) CaN_5_ and (**d**) (Ca,Li)N_5._

The refined crystal structure data for samples with *x*=0, 0.2 are given in Supplementary Fig. S2 and Supplementary Tables S2 and S3. The structural refinement of the samples reveals that the crystal structure of the solid-solution phases remains unchanged, but the lattice constants and the lattice volume linearly decrease as the solubility of LiSi_2_N_3_ increases (Figure 2a and 2b). The shrinkage of *a* (1.32%) is much larger than that of the *b* (0.18%) and *c* (0.60%) constants. This leads to a total shrinkage of 2.33% of the cell volume. Furthermore, the ratio of *a*/*b*=1.708 and *c*/(*b*/√3)=1.542 for *x*=0.20 implies that the sample has a structure that is remarkably distorted from the ideal wurtzite structure (*a*/*b*=1.732, *c*_H_/*a*_H_=1.633). Conversely, the average Ca(Eu)–N distance unusually increases with increasing LiSi_2_N_3_ content to 2.6035 and 2.6179 Å for *x*=0 and 0.2, respectively (Figure 2c). It thus leads to an expanded (Ca, Li, Eu)N_5_ polyhedron that may influence the photoluminescence of the solid solution phosphors, as schematically shown in Figure 3d. The increased bond length of Ca(Eu)–N may be ascribed to the substitution of larger Ca^2+^ ions by smaller Li^+^ ones.

Interestingly, the (Si,Al)–N distances reduce linearly with the substitution, which is ascribed to the increased Si/Al ratio (1.0 for *x*=0 and 1.5 for *x*=0.2) as the LiSi_2_N_3_ content increases (Figure 2d). Moreover, the Ca–(Si,Al) distance also becomes slightly smaller after the substitution. It varies in the range of 3.1589–3.4404 Å (3.2689 Å in average) for *x*=0 and of 3.1976–3.3885 Å (3.2680 Å in average) for *x*=0.2. Both reductions in (Si,Al)–N and Ca–(Si,Al) distances are indicative of the shrinkage of the second coordination sphere (Figure 3b), which may affect the thermal quenching of the solid solution phosphors^28^.

### Structural characterizations

**Figure 4:**
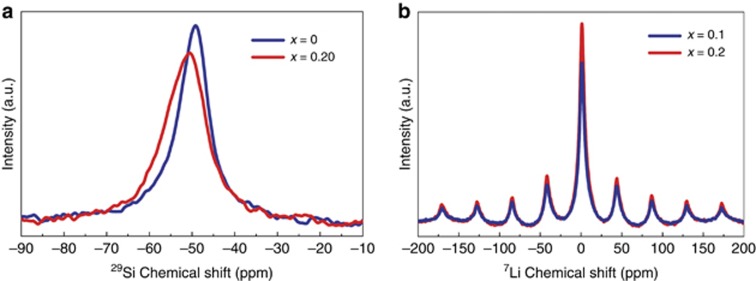
^29^Si (**a**) and ^7^Li (**b**) solid state NMR spectra of Ca_1__−__*x*_Li*_x_*Al_1__−__*x*_Si_1+*x*_N_3_:Eu^2+^ with different compositions.

Solid state NMR spectroscopy provides a very precise characterization of the local arrangement around atoms. As shown in Figure 4a, the ^29^Si isotropic chemical shifts for both compositions are found to lie in a very narrow range, from *δ*_Si_=−49 to *δ*_Si_=−50.5 ppm, indicating that the silicon environments in all solid solution phases are electronically similar and that Si is tetrahedrally coordinated with N^29^. Moreover, the composition of *x*=0.2 exhibits a resonance signal with a reduced intensity, broadened spectrum and negatively shifted peak, suggesting the enhanced Si–Al structural disorder.

The ^7^Li-MAS NMR spectra of both samples (*x*=0.1, 0.2) consist of a single intensified line at 1.08 ppm with a wide sideband pattern. The ^7^Li chemical shift has been reported as 1.3 ppm for LiSi_2_N_3_ (ref. 30). The small chemical shift difference between Ca_1__−_*_x_*Li*_x_*Al_1__−_*_x_*Si_1+*x*_N_3_ and LiSi_2_N_3_ suggests that the local environments of Li^+^ are geometrically similar. The presence of ^7^Li signals also evidences the dissolution of LiSi_2_N_3_ into CaAlSiN_3_.

The valence state of europium was investigated by analyzing the Eu L_3_ XANES spectra of solid solution phosphors. EuCl_2_ and Eu_2_O_3_ were used as reference samples for labeling the position of Eu^2+^ (6973.6 eV) and Eu^3+^ (6982.1 eV), respectively. As seen in Supplementary Fig. S3, all of the spectra show a dominant Eu^2+^ peak at ~6973.0 eV and a Eu^3+^ shoulder at ~6981 eV, indicating the coexistence of Eu^2+^ and Eu^3+^ in all samples^31^. The Eu^2+^/Eu^3+^ ratio, however, slightly increases as LiSi_2_N_3_ is accommodated into CaAlSiN_3_. This increment may have a positive influence on the photoluminescence and thermal stability of phosphors, as reported for Sr_2_Si_5_N_8_:Eu^2+^ by Yeh *et al*^32^.

### Electronic and band structures

The band gaps of the Ca_1__−__*x*_Li*_x_*Al_1__−__*x*_Si_1+x_N_3_ samples were determined from their diffuse reflection spectra. As shown in Supplementary Fig. S4, the band gap is ~4.91 eV for the *x*=0 sample and increases up to 5.08 eV for *x*=0.22. This indicates that the introduction of LiSi_2_N_3_ widens the band gap of CaAlSiN_3_ progressively, which follows Vegard’s law well, as LiSi_2_N_3_ (6.4 eV) has a larger band gap than CaAlSiN_3_^33^. The band gap of 4.91 eV is very close to the value of 5.0 eV reported by Piao *et al*^34^.

**Figure 5:**
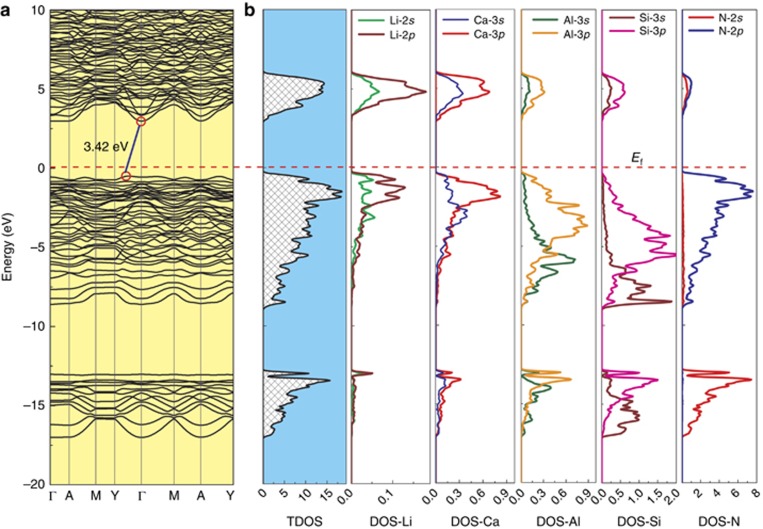
(**a**) Band structure and (**b**) total and atomic (Ca, Li, Al, Si, N) density of states of Ca_1__−_*_x_*Li*_x_*Al_1__−_*_x_*Si_1+*x*_N_3_ (*x*=0.20).

The band structure of the phosphors was calculated via first-principles using VASP^26, 27^. As illustrated in Figure 5a, the band structure of the *x*=0. 2 sample shows an indirect band gap of 3.42 eV. The top of the valence band is within the Y-Γ region, and the bottom of the conduction band is at the Γ point. The computed band gap of CaAlSiN_3_ (*x*=0) is 3.4 eV, which agrees well with the value calculated by Mikami *et al*^22^. Again, the LiSi_2_N_3_-substituted CaAlSiN_3_ has a slightly large band gap, exhibiting the same tendency as the experimental values. It is well known that the calculated band gaps are always underestimated when using the density functional theory approximation (GGA). Moreover, the atomic projected density of states indicates that the valence band consists of Ca-3*s*3*p*, Li-2*s*2*p*, Al-3*s*3*p*, Si-3*s*3*p* and N-2*s*2*p* states, varying from −17 eV to the Fermi level (*E*_f_). N-2*p*, Al-3*p* and Si-3*p* are the dominated states and are largely hybridized on the top of the valence band (−9–0 eV), with the band width of the 2*p* states of N and the 3*p* states of Al and Si being ~9 eV, which is similar to Si_3_N_4_, implying that the strong covalent bonding of (Si,Al)–N forms. Within lower energy ranges (<−13 eV), N-2*s*, Si-3*s* and Al-3*s*, as well as Ca-3*s*, are dominant, and the contribution of Li-2*p* is very limited. Moreover, they have an energy gap of ~3.9 eV with the top of the valence band. At the upper part of the valence band from −0.25 eV to the Fermi level, the N-2*p* states are mainly hybridized with Ca-3*p* and Li-2*p* states, which are expected to form chemical bonds between them. The conduction band distributes between 2.95–6 eV, in which Ca-3*s*3*p* states are at the bottom of the conduction band.

### Simultaneous spectral broadening and blueshift

In Supplementary Fig. S5, it is obvious that the emission of phosphors changes from deep red for *x*=0 to orange for *x*=0.22 under 365 nm excitation, indicating a significant blueshift in the emission spectra associated with the accommodation of LiSi_2_N_3_. Both external and internal quantum efficiencies of the samples slightly decrease as the LiSi_2_N_3_ content increases. As shown in Supplementary Fig. S6, the external quantum efficiency is 71 and 61% for *x*=0 and 0.20 upon 450 nm excitation, respectively.

**Figure 6:**
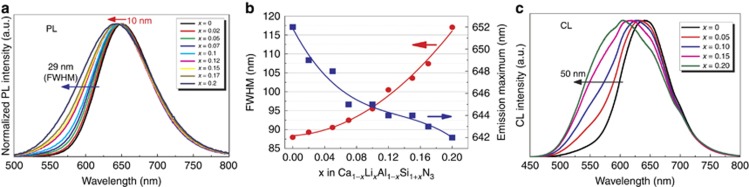
Emission spectra of Ca_1__−_*_x_*Li*_x_*Al_1__−_*_x_*Si_1+*x*_N_3_ (1.0 mol% Eu^2+^). (**a**) Photoluminescence emission bands, (**b**) the FWHM and emission maximum and (**c**) cathodoluminescence spectra.

The emission spectra, measured under 450 nm excitation, demonstrate an obvious spectral broadening (Figure 6a and Supplementary Fig. S7). The spectrum broadens as the left wing is enhanced and blueshifted, whereas the right wing remains almost unchanged. As shown in Figure 6b, the FWHM increases monotonically from 88 nm (*x* =0) to 117 nm (*x*=0.20). The large band width achieved at high LiSi_2_N_3_ concentrations is very exceptional for a single Eu^2+^ center. It is usually ~90 nm in most hosts and even remarkably narrowed when Eu^2+^ resides at a highly symmetric site (for example, SrLiAl_3_N_4_:Eu^2+^~50 nm; β-sialon:Eu^2+^~55 nm)^12, 35^. The spectral width is exclusively dependent on the local environment surrounding Eu^2+^, including the site symmetry, coordination number, structural disorder and composition fluctuation. The anomalous spectral broadening of Ca_1__−_*_x_*Li*_x_*Al_1__−_*_x_*Si_1+*x*_N_3_:Eu^2+^ can be attributed to (i) reduced structure symmetry, (ii) enhanced structural disorder and (iii) statistical composition fluctuation caused by the random distribution of Al and Si at the same crystallographic site.

Effects of both structural disorder and compositional fluctuation on the spectral broadening are also evidenced by measuring the CL spectrum of Ca_1__−_*_x_*Li*_x_*Al_1__−_*_x_*Si_1+*x*_N_3_:Eu^2+^. As seen in Figure 6c, the CL spectrum becomes inhomogeneous and again broadens with increasing LiSi_2_N_3_ content. The FWHM is significantly increased from 91 nm (*x*=0) to 141 nm (*x*=0.2). Because the nitrogen concentration is almost unchanged in all samples, the variation of the Si/Al ratio hence plays a crucial role in the change of the spectral shape, as well as the spectral width. Analyzed via Gaussian fitting, the CL spectrum was divided into two sub-bands peaking at 590 nm (Eu_I_) and 650 nm (Eu_II_), respectively (Supplementary Fig. S8). Eu_I_ is believed to reside at a Si-rich site with a longer Eu_I_–Si distance, whereas Eu_II_ resides at an Al-rich site with a shorter Eu_II_–Al distance because Si^4+^ has a smaller ionic size than Al^3+^. The luminescence intensity ratio of Eu_I_/Eu_II_ for compositions with *x*=0, 0.05, 0.10, 0.15 and 0.20 is, respectively, 0.19, 0.45, 0.73, 1.27 and 1.80, which is in a good agreement with the enhanced left wing and broadened spectra.

In addition to the spectral broadening, the blueshift of both emission and excitation spectra is also an interesting feature of the solid solution phosphors (Figure 6a and Supplementary Fig. S7). The peak emission is blueshifted by 10 nm, moving from 652 nm (*x*=0) to 642 nm (*x*=0.20). It is already known that both lattice parameters and the cell volume reduce after the introduction of LiSi_2_N_3_ and that the lattice shrinkage usually yields large crystal field splitting, which may redshift the photoluminescence spectra. However, this is not the case in this work. Besides the influence of the overall lattice volume, the luminescence of Eu^2+^ is much more affected by the local environment and band-gap structure. The unexpected blueshift is therefore attributed to (i) an enlarged Ca(Eu)N_5_ polyhedron that actually decreases the crystal field strength, (ii) an expanded band gap that lifts up the lowest position of the Eu^2+^ 5*d* state and (iii) an enhanced Eu_I_ emission intensity that increases the left wing of the emission band.

The blueshift of the deep-red CaAlSiN_3_:Eu^2+^ is desired for achieving high-luminous efficacy because the emission spectrum will be much closer to the eye sensitivity curves. Its emission band can be dramatically blueshifted via the Sr substitution for Ca, but the band width is significantly narrowed simultanously^11^. As a result, the color rendering indices are sacrificed to obtain higher luminous efficacy when using (Sr,Ca)AlSiN_3_:Eu^2+^. To avoid such a loss in color rendition, spectral broadening is simultaneously requested to compensate the reduction of the red spectral component. Interestingly and fortunately, Ca_1__−_*_x_*Li*_x_*Al_1__−_*_x_*Si_1+*x*_N_3_:Eu^2+^ shows both spectral broadening and blueshifting of the emission band, thus enabling it to be a very promising red phosphor for realizing high-luminous efficacy without sacrificing the color rendition.

### Substantial enhancement in thermal quenching

**Figure 7:**
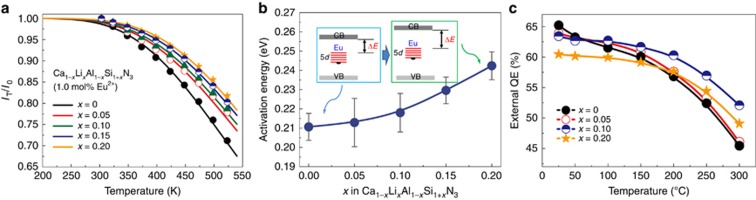
Thermal stability of Ca_1__−_*_x_*Li*_x_*Al_1__−_*_x_*Si_1+*x*_N_3_ (1.0 mol% Eu^2+^). (**a**) Temperature-dependent emission intensity. The solid lines were plotted by substituting the fitted *E*_a_ into the Arrhenius equation. (**b**) Activation energy *E*_a_ for thermal quenching. The inset shows the schematic band structure and the energy levels of 5*d* of Eu^2+^. (**c**) Temperature-dependent quantum efficiency.

The heat generated in LED chips, sometimes higher than 100 °C, will definitely induce luminescence quenching or even degrade phosphors; thus, the phosphors must have small thermal quenching to sustain the long lifetime of LED devices. The temperature-dependent luminescence shown in Figure 7a indicates that thermal quenching is progressively reduced with increasing LiSi_2_N_3_ content. At 150 °C (~423 K), the emission intensity is reduced by 12% for *x*=0 but only by 6% for *x*=0.20. The difference in thermal stability is more pronounced at higher temperatures, showing a decline of 29% for *x*=0 and 18% for *x*=0.20 at 250 °C (~523 K). The emission intensity was fitted according to the Arrhenius equation *I*_T_/*I*_0_=[1+*A* × exp(−*E*_a_/*kT*)]^−1^, where *I*_0_ and *I*_T_ are, respectively, the intensities at absolute zero and temperatures of 25–250 °C (298–523 K), *A* is a constant, and *E*_a_ is the energy barrier for thermal quenching^36^. *E*_a_ is shown to increase from 0.21 eV (*x*=0) to 0.24 eV (*x*=0.20), indicating a higher thermal barrier for luminescence quenching after the LiSi_2_N_3_ substitution (Figure 7b). Such an improvement in thermal stability was also observed by Wang and colleagues^37^.

Moreover, the temperature-dependent quantum efficiency also has a similar tendency, showing a smaller thermally-induced reduction for the solid solution phosphors (Figure 7c). Although the introduction of LiSi_2_N_3_ reduces the efficiency slightly at room temperature (that is, from 65 to 61% under 450 nm excitation), it obviously retards the thermal quenching. At 300 °C, the external quantum efficiency is reduced by 33.3% for *x*=0 and by 19.6% for *x*=0.2. This enables the broadband Ca_1__−_*_x_*Li*_x_*Al_1__−_*_x_*Si_1+*x*_N_3_:Eu^2+^ (*x*=0.2) to be superior in thermal stability to (Ca_1__−_*_x_*Sr*_x_*)AlSiN_3_:Eu^2+^ when the Sr substitution does not change the thermal quenching.

Thermal quenching of a phosphor greatly depends on the electronic and band structures of host crystals, that is, on the crystallographic site where Eu^2+^ resides and the position where the 5*d* state of Eu^2+^ is in between the band gap. As mentioned, the band gap widens due to the substitution of [Li,Si]^5+^ for [Ca,Al]^5+^ in CaAlSiN_3_. This enlargement separates the distance (Δ*E*) between the highest position of the 5*d* state of Eu^2+^ and the bottom of the conduction band to a larger degree, leading to an increased activation energy for thermal quenching and therefore minimizing the photoionization (Figure 7b). Moreover, as addressed by Liu and colleagues^17^, the second coordination sphere also yields an effect on the luminescence quenching. With LiSi_2_N_3_ dissolving in the CaAlSiN_3_ lattice, the second coordination sphere (that is, Eu[Si/Al]*_n_* polyhedron) is constrained with an increasing Si^4+^/Al^3+^ ratio (from 1.0 to 1.5), counteracting the expansion of the first coordination sphere. This will result in a decrease of the non-radiative losses in the luminescence process and hence higher quenching temperatures. Wang and colleagues^37^ proposed a remote control effect to interpret the enhanced thermal quenching as Li partially substitutes Ca in CaAlSiN_3_.

### High efficiency and color rendering white LEDs

(Sr,Ca)AlSiN_3_:Eu^2+^ is an excellent short-wavelength red phosphor commonly used in highly efficient wLEDs, but its narrow emission band leads to a medium color rendering index Ra and a very low R9 index. The solid solution phosphors Ca_1__−_*_x_*Li*_x_*Al_1__−_*_x_*Si_1+*x*_N_3_:Eu^2+^ developed in this work exhibit both broadened and blueshifted emission spectra, enabling the simultaneous achievement of high-luminous efficacy and color rendition wLEDs. To verify this, two compositions, with *x*=0 (RD1, *λ*_em_=652 nm, FWHM=88 nm) and *x*=0.20 (RD2, *λ*_em_=642 nm, FWHM=117 nm), were chosen as red phosphor to fabricate wLEDs and compared with a commercial (Sr,Ca)AlSiN_3_:Eu^2+^ phosphor (RD3, *λ*_em_=630 nm, FWHM=84 nm). RD1 and RD2 were obtained by annealing the as-synthesized phosphors at 1800 °C for 4 h; their particle morphologies are given in Supplementary Fig. S9. Both samples show an identical primary particle size of ~5 μm. The external quantum efficiency of RD1 and RD2 is, respectively, increased to 78 and 70%, both being still smaller than 82% for RD3.

**Figure 8:**
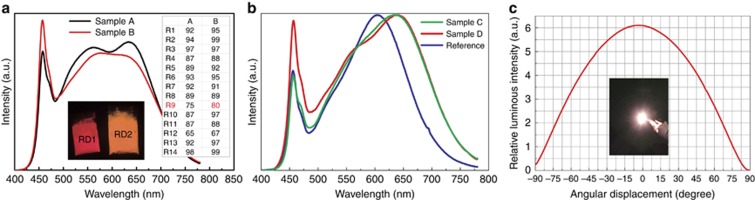
Electroluminescence spectra of wLEDs using different combinations of red and green phosphors. (**a**) Three-phosphor-converted wLEDs, (**b**) two-phosphor-converted wLEDs and (**c**) spatial radiation pattern and photograph of Sample D.

The type I white LEDs (samples A and B) were prepared by combining RD1/RD2 with a green (Ga,Y)_3_Al_5_O_12_:Ce^3+^ (G1, *λ*_em_=535 nm) and a blue BaSi_2_O_2_N_2_:Eu^2+^ (B1, *λ*_em_=490 nm) phosphor. As shown in Figure 8 and Table 1, white LEDs using RD2 generally have higher color rendering indices (for example, Ra and R9) than those using RD1. This indicates that the color rendition is not sacrificed due to the spectral blueshift in RD2 but compensated greatly by the spectral broadening. The luminous efficacy of the device using RD2 is 7% lower than that using RD1, which is attributable to the smaller quantum efficiency of RD2 (8% lower). It is believed that the luminous efficacy will be the same even if the processing conditions of RD2 are further optimized.

For comparison with the commercial red phosphor (RD3), Type II wLEDs (samples C and D) were fabricated by combining RD2/RD3 with a green phosphor (G1). As shown, the color rendering index using RD3 (Ra=84 and R9=21) is remarkably lowered, typically the R9 index, due to its narrowed and blueshifted emission band. This again demonstrates the trade-off between color rendering index and luminous efficacy by using common red phosphors. However, a higher color rendering index (Ra=92, R9=69) can be maintained without compromising too much luminous efficacy when using RD2. The relatively low luminous efficacy (103 *vs* 123 lm W^−1^) is actually again due to the low quantum efficiency of RD2. Much higher color rendering indices of Ra=95 and R9=96, as well as a high luminous efficacy of 101 lm W^−1^, are obtained by combining RD2 with a short-wavelength Lu_3_Al_5_O_12_:Ce^3+^ green phosphor (G2, *λ*_em_=525 nm). This highlights the point that a good balance between the luminous efficacy and color rendition occurs when utilizing the broadband and blueshifted red phosphor discovered in the current work.

## Conclusions

Spectral tuning of phosphors is of great importance to control or optimize the optical properties of solid state lighting devices. In this work, by applying the structure and band-gap engineering strategies, we have developed a promising Ca_1-*x*_Li*_x_*Al_1-*x*_Si_1+*x*_N_3_:Eu^2+^ solid solution red phosphor with simultaneous spectral broadening and blueshift and solved the essential trade-off between luminous efficacy and color rendition. High-performance wLEDs with super-high color rendering indices of Ra=96 and R9=95 and a preserved luminous efficacy of 101 lm W^−1^ have been attained by using the broadband red phosphor. Due to the constrained second coordination sphere and widened band gap, a substantial improvement in thermal quenching has also been obtained after LiSi_2_N_3_ substitution. This new broadband red phosphor is superior in color quality and thermal stability to red phosphors, such as (Ca_1__−_*_x_*Sr*_x_*)AlSiN_3_:Eu^2+^ and K_2_SiF_6_:Mn^4+^, currently used for general lighting.

## Figures and Tables

**Table 1:** Color rendering properties (Ra and R9), luminous efficacy (*η*) and color temperatures (CCT) of wLEDs

Samples	Phosphors	Ra	R9	*η* (lm W^−1^)	CCT (K)
A	RD1+G1+B1	91	75	100	3971
B	RD2+G1+B1	93	80	93	4278
C	RD2+G1	92	69	103	2973
D	RD2+G2	95	96	101	3036
Ref.	RD3+G1	84	21	123	3519
