# Supplementary material for: Ca1−xLixAl1−xSi1+xN3:Eu2+ solid solutions as broadband, color-tunable and thermally robust red phosphors for superior color rendition white light-emitting diodes
Source: Light Sci Appl. 2016 Oct 21;5(10):e16155–. doi: 10.1038/lsa.2016.155 (PMC6059829; doi:10.1038/lsa.2016.155)
Supplement: Supplementary Information [file lsa2016155x1.doc]

**Supplementary Information for**

**Ca_1-_*_x_*Li*_x_*Al_1-_*_x_*Si_1+_*_x_*N_3_:Eu^2+^ solid solutions as broadband, color-tunable and thermally robust red phosphors for superior color rendition white light-emitting diodes**

**Le Wang,^1^ Rong-Jun Xie,^2,3^ Yuanqiang Li,^4^ Xiaojun Wang,^3^ Chong-Geng Ma,^5^ Dong Luo,^1^ Takashi Takeda,^3^ Yi-Ting Tsai,^6^ Ru-Shi Liu,^6,7^ Naoto Hirosaki^3^**

1. College of Optics and Electronic Science and Technology, China Jiliang University, Hangzhou, Zhejiang 310018, China

2. College of Materials, Xiamen University, Xiamen, Fujian 361005, China

3. Sialon Group, National Institute for Materials Science (NIMS), Tsukuba, Ibaraki 305-0044, Japan

4. Intematix Corporation, Fremont, CA 94538, USA

5. College of Sciences, Chongqing University of Posts and Telecommunications, Chongqing 400065, China

6. Department of Chemistry, Taiwan University

7. Department of Mechanical Engineering and Graduate Institute of Manufacturing Technology, Taipei University of Technology

Correspondence: Rong-Jun Xie, Email: rjxie@xmu.edu.cn; Le Wang, Email: calla@cjlu.edu.cn

**ICP**

Table S1 Chemical compositions of Ca_1-_*_x_*Li*_x_*Al_1-_*_x_*Si_1+_*_x_*N_3_:Eu^2+^ (mol%)

|  | Ca | Li | Eu | Al | Si | N | O |
| --- | --- | --- | --- | --- | --- | --- | --- |
| ***x* = 0** | 28.4  28.7 | 0.0  0 | 1.0  1.1 | 19.5  19.5 | 19.8  20.3 | 29.4  30.4 | 1.9  0 |
| ***x* = 0.05** | 27.5  27.6 | 0.1  0.3 | 1.1  1.1 | 18.6  18.8 | 21.0  21.6 | 29.7  30.7 | 1.9  0 |
| ***x* = 0.10** | 26.0  26.4 | 0.2  0.5 | 1.2  1.1 | 17.9  18.0 | 22.3  22.9 | 30.0  31.1 | 1.8  0 |
| ***x* = 0.15** | 24.9  25.2 | 0.4  0.8 | 1.1  1.1 | 17.1  17.2 | 23.6  24.2 | 30.4  31.5 | 1.8  0 |
| ***x* = 0.20** | 23.4  24.0 | 0.6  1.1 | 1.2  1.2 | 16.0  16.4 | 25.6  25.6 | 30.7  31.9 | 1.9  0 |

* The data in red are the nominal chemical compositions of samples.

**Figure S1** Calculated lattice energy as a function of unit cell volume with the orthorhombic and monoclinic structure for Ca_1-_*_x_*Li*_x_*Al_1-_*_x_*Si_1+_*_x_*N_3_:Eu^2+^ (*x* = 0.20).

**Figure S2** Measured and calculated XRD patterns of Ca_1-_*_x_*Li*_x_*Al_1-_*_x_*Si_1+_*_x_*N_3_:Eu^2+^ with the composition of (a) *x* = 0, and (b) *x* = 0.20. The sample of *x* = 0.20 shows a little amount of AlN impurity.

Table S2 Atomic coordination of Ca_1-_*_x_*Li*_x_*Al_1-_*_x_*Si_1+_*_x_*N_3_: 1 mol% Eu^2+^ (*x* = 0.20)

| Atom | x | y | z | Occupancy | Ui/Ue |
| --- | --- | --- | --- | --- | --- |
| Ca1 | 0 | 0.32994(22) | 0.4955(4) | 0.79 | 0.0163 |
| Li1 | 0 | 0.32994(22) | 0.4955(4) | 0.20 | 0.0163 |
| Eu1 | 0 | 0.32994(22) | 0.4955(4) | 0.01 | 0.0163 |
| Al1 | 0.17414(11) | 0.15240(17) | 0.020156 | 0.40 | 0.0078 |
| Si1 | 0.17414(11) | 0.15240(17) | 0.020156 | 0.60 | 0.0078 |
| N1 | 0.21736(24) | 0.1267(5) | 0.3756(6) | 1.00 | 0.0014 |
| N2 | 0 | 0.2326(6) | -0.0082(10) | 1.00 | 0.0084 |

* The R-factors of the refinements are R_wp_ = 7.72%, R_p_ = 5.82%, χ^2^ = 3.12

Table S3 Selected bond length (Å) of Ca_1-_*_x_*Li*_x_*Al_1-_*_x_*Si_1+_*_x_*N_3_:1 mol% Eu^2+^ (*x*=0.2)

| (Ca, Li, Eu)1-N1 | 2.4630(22) | (Si,Al)1-N1 | 1.8380(30) |
| --- | --- | --- | --- |
| (Ca, Li, Eu)1-N1 | 2.4630(22) | (Si,Al)1-N1 | 1.7832(26) |
| (Ca, Li, Eu)1-N2 | 2.588(4) | (Si,Al)1-N1 | 1.7815(25) |
| (Ca, Li, Eu)1-N2 | 2.551(4) | (Si,Al)1-N2 | 1.7442(14) |
| (Ca, Li, Eu)1-N2 | 3.1740(32) |  |  |
| (Ca, Li, Eu)1-N2 | 2.4679(32) |  |  |
| Average | 2.6178(19) | Average | 1.7867(23) |

**Figure S3** Eu L_3_ XANES spectrum of Ca_1-_*_x_*Li*_x_*Al_1-_*_x_*Si_1+_*_x_*N_3_:Eu^2+^ with *x* = 0, 0.07 and 0.20.

**Figure S4** Diffuse reflection spectra of samples with (a) *x* = 0, (b) *x* = 0.10, (c) *x* = 0.15, and (d) *x* = 0.22. The black and red curves are blank and doped samples, respectively.


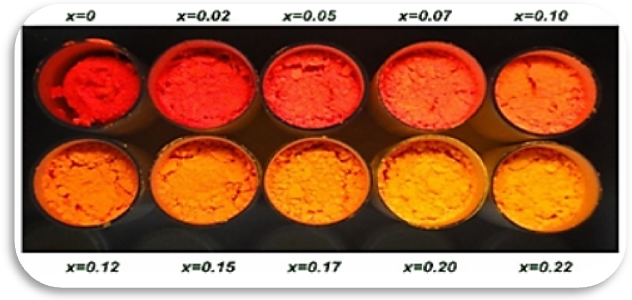


**Figure S5** Photographs of as-synthesized samples with varying LiSi_2_N_3_ under the UV light irradiation.

**Figure S6** External and internal quantum efficiencies of Ca_1-_*_x_*Li*_x_*Al_1-_*_x_*Si_1+_*_x_*N_3_:Eu^2+^ (*x* = 0 – 0.20). The Eu concentration is 1 mol%.

**Figure S7** Excitation (a) and emission (b) spectra of Ca_1-_*_x_*Li*_x_*Al_1-_*_x_*Si_1+_*_x_*N_3_:Eu^2+^. The excitation spectra were obtained by monitoring 650 nm, and the emission spectra were measured under 450 nm excitation.

**Figure S8** Gaussian fitting of the emission spectra of Ca_1-_*_x_*Li*_x_*Al_1-_*_x_*Si_1+_*_x_*N_3_:Eu^2+^ (a) *x* = 0, (b) *x* = 0.05, (c) *x* = 0.1, (d) *x* = 0.15, and (e) *x* = 0.2. The blue dotted lines are fitted two emission bands of the sample, and the red dotted curve is the fitted emission spectrum.


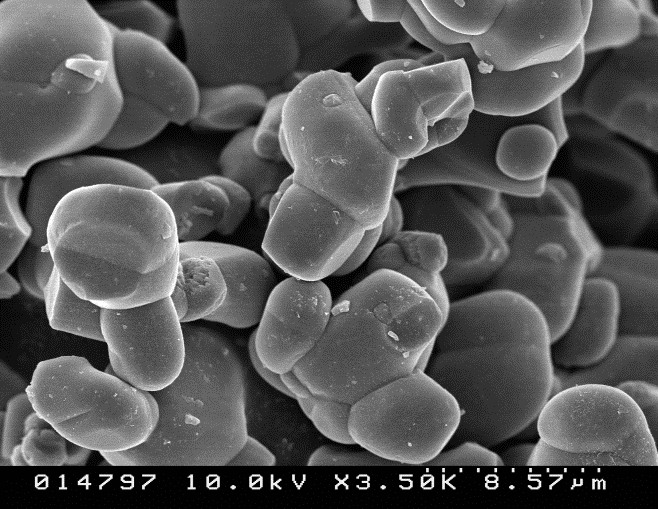

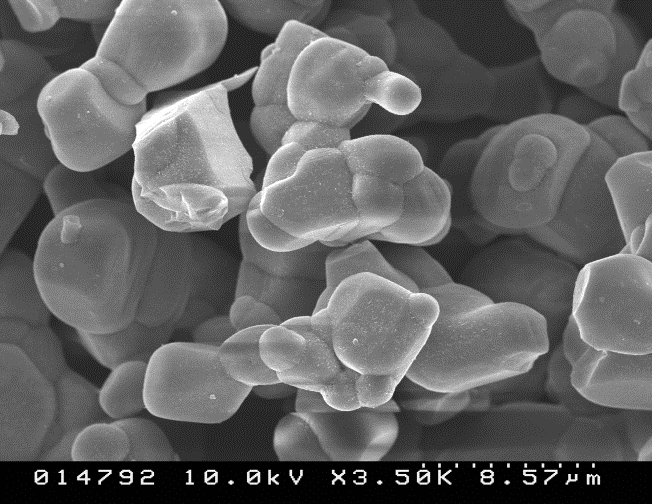


**Figure S9** SEM images of Ca_1-_*_x_*Li*_x_*Al_1-_*_x_*Si_1+_*_x_*N_3_:Eu^2+^ (upper, *x* = 0; and lower, *x* = 0.2) after the post-treatment in water.
